# Significance of Crosslinking Approaches in the Development of Next Generation Hydrogels for Corneal Tissue Engineering

**DOI:** 10.3390/pharmaceutics13030319

**Published:** 2021-02-28

**Authors:** Promita Bhattacharjee, Mark Ahearne

**Affiliations:** 1 Trinity Centre for Biomedical Engineering, Trinity Biomedical Sciences Institute, Trinity College Dublin, University of Dublin, D02 R590 Dublin, Ireland; promitabhatt@gmail.com; 2 Department of Mechanical, Manufacturing and Biomedical Engineering, School of Engineering, Trinity College Dublin, University of Dublin, D02 R590 Dublin, Ireland

**Keywords:** cornea, hydrogel, keratoplasty, scaffold, tissue engineering, collagen

## Abstract

Medical conditions such as trachoma, keratoconus and Fuchs endothelial dystrophy can damage the cornea, leading to visual deterioration and blindness and necessitating a cornea transplant. Due to the shortage of donor corneas, hydrogels have been investigated as potential corneal replacements. A key factor that influences the physical and biochemical properties of these hydrogels is how they are crosslinked. In this paper, an overview is provided of different crosslinking techniques and crosslinking chemical additives that have been applied to hydrogels for the purposes of corneal tissue engineering, drug delivery or corneal repair. Factors that influence the success of a crosslinker are considered that include material composition, dosage, fabrication method, immunogenicity and toxicity. Different crosslinking techniques that have been used to develop injectable hydrogels for corneal regeneration are summarized. The limitations and future prospects of crosslinking strategies for use in corneal tissue engineering are discussed. It is demonstrated that the choice of crosslinking technique has a significant influence on the biocompatibility, mechanical properties and chemical structure of hydrogels that may be suitable for corneal tissue engineering and regenerative applications.

## 1. Introduction

The cornea is the outermost transparent layer of the anterior eye consisting of five distinct layers: Epithelium, Bowman’s layer, Stroma, Descemets membrane and Endothelium. Damage to any of these layers can result in a loss of vision. More than 10 million people worldwide suffer from corneal related blindness due to disease or injury [1]. Corneal blindness can result from infection, inflammation, trauma, dystrophies and degenerative medical conditions. Partial or full corneal transplantation (keratoplasty) is often the only viable treatment to regain vision. However, some of the problems associated with keratoplasties include immunological rejection (around 18%) [2] and donor shortages [3,4].

An alternative to traditional keratoplasty is to develop an artificial cornea or keratoprosthesis. This approach has the advantage of overcoming the donor supply problems associated with keratoplasties. However, current keratoprostheses have a number of limitations including an increased risk of glaucoma, inflammation and abnormal tissue growth [5]. Amniotic membrane (AM) obtained from the inner wall of the fetal placenta has been used for ocular surface reconstruction [6,7]. The AM promotes re-epithelialization of the corneal surface, reduces inflammation and inhibits vascularization [8]. However, using AM to reconstruct the ocular surface has drawbacks, including reduced transparency [9], poor mechanical strength [10] and varying tissue quality between donors [11].

To overcome these problems, tissue engineering approaches have been under investigation to fabricate whole corneas or specific layers of the cornea that are suitable for transplantation. These may be generated using decellularized xenogenic tissues [12,13,14,15] or natural or synthetic polymers [16,17,18,19], as a scaffold to support cells in a three-dimensional construct. To engineer a functional corneal equivalent, constructs should ideally mimic the native cornea, both structurally and functionally. Tissue engineered corneas need to exhibit three functional characteristics: protection, light transmission, and refraction [20]. To fulfill these characteristics, constructs should support the development of a functional corneal epithelium by supporting proliferation and migration of cells from the limbus. This newly formed epithelium should protect the intra-ocular contents from pathogenic invasion. The mechanical stiffness and strength of the constructs should be equivalent to the native cornea. Ideally, the constructs should mimic the nanoscale fibrillar structure of the corneal stroma to achieve a high degree of transparency (>90%). To prevent the formation of an optical haze, the construct’s swelling ratio should be similar to the native cornea. Engineered corneal equivalents should also have a high water content to allow nutrient diffusion through the tissue, enhance cell survival and replicate the cornea’s viscoelastic characteristics.

**Figure 1:**
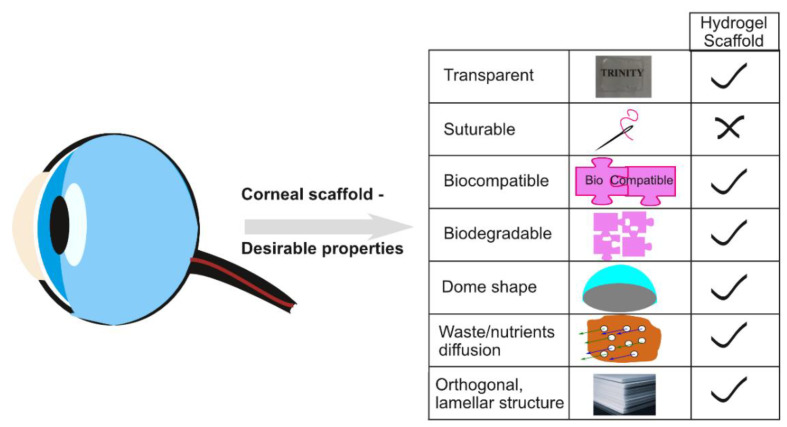
Schematic representation of desirable properties that hydrogels should possess for corneal tissue engineering.

Hydrogels are water-swollen polymers that have been under investigation as scaffolds to engineer corneal tissue for many reasons including their high water content, biocompatibility, transparency and permeability (Figure 1). While many hydrogels tend not to be suturable, some can adhere directly to tissue when gelation occurs in vivo, avoiding the need for sutures [21]. Hydrogels can also be used to deliver drugs to the eye to support tissue regeneration and inhibit inflammation. They offer many advantages over colloidal [22,23] and polymeric [24] drug delivery systems including a high water content that assists in preserving the activity of bio-pharmaceuticals such as peptides, proteins or nucleic acids [25,26]. Temperature-responsive and in situ chemically crosslinked hydrogels can be administered by minimally invasive methods [27,28,29]. While hydrogels have been shown to support the formation of the functional epithelium [30], many have poor mechanical strength and rigidity compared to native corneas [31], they can undergo rapid degradation in vivo and they often lack signaling molecules normally resident in the extracellular matrix that are necessary to control cell behavior. 

To assist in improving the mechanical and degradation characteristics of hydrogels, the application of exogenous small molecules, i.e., crosslinkers [28] has been investigated. Crosslinking agents have been introduced to functionally modify the mechanical, biological and degradation properties of various biomaterials depending on their compositional and structural features [32]. It is important to select a suitable crosslinker for specific tissue applications that allows the possibility to tune the hydrogels micro/macro-structure and physico-chemical, biological and mechanical properties. 

While many different types of crosslinkers have been investigated for controlling the properties of hydrogels for corneal tissue engineering and regeneration, these have not been previously compared in any detail. Here, we report on the recent investigations involving the functional modification of hydrogels using different crosslinking reagents. This paper reviews different crosslinking approaches that have been employed to fabricate several standard and innovative hydrogels for corneal regeneration. The basic mechanisms of each crosslinking method are described and examples are used to illustrate each of the approaches. Several studies are highlighted that have undertaken comparative analyses of different crosslinking reagents. The development of injectable hydrogels and the impact of different crosslinking initiators on the characteristics of hydrogels are discussed. The benefits, limitations and future prospects of these crosslinkers used for corneal regeneration are outlined.

## 2. Crosslinking in Hydrogel Fabrication for Corneal Regeneration 

**Figure 2:**
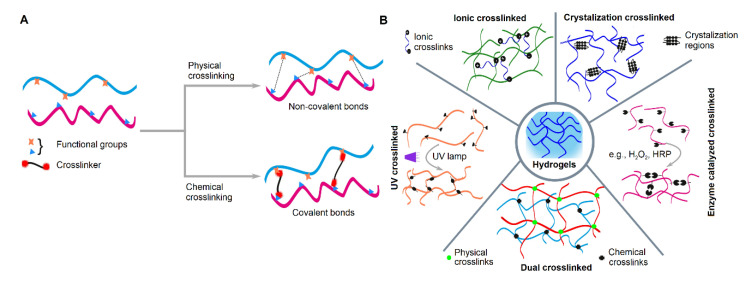
Representation showing (**A**) the effect of physical and chemical crosslinking on the type of bonds formed and (**B**) several examples of different crosslinking techniques.

Recently, there has been much progress in fabricating mechanically stable biomimetic scaffolds and hydrogels by incorporating different crosslinking mechanisms. Crosslinking is an important parameter in the fabrication of hydrogels that can result in enhanced biomechanical properties by developing inter-molecular network linkages. Among the different major functional groups (hydroxyl, methyl, carbonyl, carboxyl, amino, phosphate, and sulfhydryl) of a polymer chain, any two functional groups can couple covalently or non-covalently through crosslinking. These types of bonds (especially covalent bonding) regulate the protein activity, stability and complex structural assembly within fabricated biomaterials [29]. Ideally, crosslinking agents should be capable of improving mechanical strength and stiffness, must be non-toxic, enhance enzymatic resistance, effectively influence cross-talk between cells and material, and retain shape memory [33]. The specific chemical and structural properties of a hydrogel have a significant impact on the crosslinking mechanism. These crosslinking mechanisms can be classified into two groups: physical involving non-covalent bonding or chemical involving covalent bonding (Figure 2A). For hydrogels, physical crosslinking is accompanied by chemical crosslinking since physical crosslinking alone would be insufficient to maintain the integrity of the hydrogel. Specific examples of crosslinking techniques are shown (Figure 2B). 

### 2.1. Dehydrothermal Treatment (DHT)

During DHT, the hydrogel is exposed to an elevated temperature under vacuum. Intermolecular crosslinking is initiated via esterification or amide formation when water molecules are evacuated at a high temperature [34]. Carboxyl and amine groups situated in adjacent proximity of protein backbone become covalently coupled. One advantage of this mechanism is that it results in sterilization of the materials, hence, removing the need for further sterilization steps later in the process as well as reducing the potential immunogenic response to the material after implantation [35,36].

An ophthalmic drug delivery system was developed using biodegradable cationized gelatin hydrogels loaded with an epidermal growth factor [37]. These hydrogels were fabricated by air-drying and DHT crosslinking. Corneal epithelial defects in rabbits were created to study the potential of this hydrogel for wound repair. A controlled release of epidermal growth factor was reported from hydrogels that led to accelerated wound healing. In a separate comparative study, DHT crosslinked gelatin hydrogel sheets and atelocollagen sheets with human corneal endothelial cells were compared, where gelatin hydrogels displayed better transparency, permeability and elasticity [38]. ZO-1 bonding between cells and Na^+^/K^+^-ATPase indicated that the crosslinked gelatin supported the formation of a functional endothelium. In another study, collagen scaffolds were crosslinked using either UV irradiation or DHT [39]. Both treatments led to increased tensile strength but also the fragmentation of the collagen molecules structure. There was no significant difference between the two mechanisms. German et al. demonstrated that they could engineer a cornea by culturing human epithelial cells on DHT crosslinked collagen hydrogels containing fibroblasts [40]. A promising result was reported with the formation of 4–5 layers of regenerated corneal epithelium as well as basement membrane components after 3 days of culture. While DHT does not induce any potential toxic effects, controlling the degree of the crosslinking remains a challenge to be addressed [41].

### 2.2. Ultra-Violet (UV) Irradiation

UV mediated crosslinking is an easy, robust and non-toxic procedure when two characteristic phenomena take place simultaneously: crosslinking and UV-induced denaturation. The combination of these two phenomena improves the mechanical properties and degradation resistance of collagen based scaffolds [42]. Protein molecules can be covalently coupled via UV light with aromatic residues such as tyrosine and phenylalanine. UV light also creates covalent bonds between polypeptide chains, important cell recognition sites situated in the proteins backbone, without involving the acidic and basic side chains [43].

UV crosslinking has been used to modify collagen based biomaterials and tissues. For example, the Young’s modulus of collagen-based hydrogels has been shown to significantly increase after UV mediated crosslinking between riboflavin and collagen without hindering the growth of human corneal fibroblasts [44,45]. The final modulus of the hydrogel was dependent on the UV exposure time. UV crosslinking also enhances enzymatic resistance in collagen hydrogels [46]. Incorporation of glucose into the hydrogel can help to lower collagen fragmentation during the UV crosslinking process [47]. Therefore, this technique could potentially be used for in vitro stabilization of collagen hydrogels [48]. UV crosslinking is also a promising technique to treat degenerative diseases such as keratoconus that directly affects the corneal stroma. In this process, photosensitive riboflavin is crosslinked with corneal collagen through UV irradiation [49]. 

In addition to collagen, silk fibroin has also been crosslinked via UV irradiation. A highly transparent silk fibroin-based hydrogel has been developed using photo-crosslinking between riboflavin and silk fibroin. This hydrogel was examined for corneal reshaping through photo-lithography to provide visual acuity. Excellent adherence between the hydrogel and ocular surface makes this approach very promising for corneal regeneration [50]. Silk fibroin based matrices also positively influence corneal stromal cell behavior when the riboflavin content and UV exposure are optimized. Riboflavin crosslinked silk fibroin matrices supported cellular adhesion, proliferation, ECM formation, and keratocyte-associated gene expression [51]. 

An injectable, photocurable and biocompatible gelatin-based thiol-acrylate hydrogel with tunable mechanical properties has been used for corneal regeneration in rabbit model [52]. This hydrogel supported epithelial wound coverage in less than three days. The study also demonstrated the non-toxic effect of UV irradiation on the cornea as well as the posterior segment of the eye [52]. Semi-synthetic gelatin methacrylate (GelMA) is a popular biomaterial in tissue engineering due to its adjustable physical properties, biocompatibility and ability to be used in 3D bioprinting. In the presence of a photoinitiator, GelMA hydrogels can be easily fabricated through free radical polymerization. To generate GelMA hydrogels suitable for corneal endothelium formation and transplantation, physical networks were formed in the solution prior to UV crosslinking by incubating a pre-polymer solution at 4 °C for 1 h [53]. The hydrogels displayed excellent in vitro biocompatibility with corneal endothelial cells and had favorable biodegradation kinetics and high cellular viability in a rabbit model following transplantation. 

In addition to natural polymer-based hydrogels, UV crosslinking can be used to produce stable hydrogels from synthetic polymers. Transparent, UV crosslinked polyethylene glycol (PEG)-diacrylate and PEG-diacrylamide hydrogels have been successfully manufactured and tested as corneal replacements in rabbit studies [54]. Although PEG-diacrylate hydrogels resulted in corneal inflammation and ulceration that led to corneal haze, PEG-diacrylamide hydrogels showed more promise. UV crosslinked PEG-diacrylamide hydrogels did not show any inflammation up to 6 months after implantation and appeared healthy and transparent. UV mediated photo-crosslinking is only effective for transparent and thin scaffolds that allow the light to penetrate the structure. For this reason, this crosslinking technique is generally acceptable and has fewer limitations for its applications with a comparatively thin tissue like the cornea.

### 2.3. Crosslinking Using Chemical Additives

Crosslinking using chemical additives accelerates the modification of the polymeric backbone and leads to a higher degree of crosslinking. For this reason, these crosslinkers are widely accepted for tissue engineering and regenerative medicine applications. The most commonly used crosslinking additives are glutaraldehyde (GA), 1,4-butanediol di glycidyl ether (BDDGE), genipin and 1-ethyl-3-[3-dimethylaminopropyl] carbodiimide hydrochloride (EDC). Examples of hydrogels crosslinked using chemical additives and that have been used for corneal regeneration are shown in Table 1. 

#### 2.3.1. Glutaraldehyde (GA)

Bi-functional crosslinking agent, GA induces covalent linkages between the aldehyde groups of GA with the amine groups of lysine or hydroxylysine residues of the polypeptide chains. This mechanism contributes to increased degradation resistivity of the protein molecules. GA is one of the most commonly used crosslinking agents due to its fast reaction time, firm stabilization, low cost and easy availability [55]. The main limitation of using GA is its cytotoxicity and the immune response it elicits in the body [56]. Unbound free aldehyde groups are mainly responsible for the toxic effect of GA crosslinked scaffolds. Vigorous washing of the crosslinked scaffold using glycine solution helps to eliminate unbound aldehyde groups reducing the scaffold’s toxicity. Several studies that have used GA crosslinked hydrogels for corneal regeneration [41,57,58,59,60,61,62,63,64,65,66,67] are summarized in Table 1. These studies show that researchers are trying to obtain a stable crosslinker by varying the concentration of GA. However, due to the toxicity of GA, alternative chemical agents had to be explored. 

#### 2.3.2. 1,4-Butanediol Diglycidyl Ether (BDDGE)

The application of BDDGE as a crosslinking agent is limited in the field of corneal tissue engineering (Table 1). BDDGE is more commonly used to efficiently stabilize collagen dermal filler [68]. The crosslinking reactivity of BDDGE with a biopolymer depends on environmental conditions such as pH and temperature. Through hydroxyl group linkage, BDDGE is also able to covalently bond with the macromolecular substrate. The crosslinking mechanism depends on the reactivity of the epoxide groups situated on the ends of the molecules. In alkaline conditions, amine groups can open up the epoxide ring, forming strong ether bonds and secondary amide bonding [68]. One example of a BDDGE crosslinked hydrogels that have been used as a bioactive corneal stromal substitute are hydroxypropyl chitosan–gelatin-chondroitin sulfate hydrogels. These hydrogels were highly transparent, retained a high water content, were permeable and showed good biocompatibility [69]. In another study, Koh et al. reported that BDDGE cross-linking of collagen hydrogels resulted in a slow gelation time [70]. The bi-functional BDDGE cross-linking exhibited through secondary amine bond formation via epoxide ring opening by amine groups of collagens under basic pH conditions. This phenomenon facilitates slower gelation and enables drug molecule encapsulation within the collagen matrix for therapeutic application.

#### 2.3.3. Genipin

Genipin is a green colored chemical derived from gardenia fruits that enables protein macromolecules to be easily crosslinked via intra- and inter-molecular linkages. This crosslinking mechanism undergoes the following two steps. First, a nucleophilic substitution takes place at C_3_ carbon atom of genipin. This leads to the immediate formation of an intermediate aldehyde group. Next, a heterocyclic compound is formed due to the reaction between the aldehyde group and secondary amine. Subsequently, the substitution of ester groups takes place on the protein backbone via secondary amide bridging and leads to nucleophilic substitution [71]. Consequently, a heterocyclic compound (bluish-green in color) is formed due to the reaction between genipin and protein amine groups via oxygen-radical-induced polymerization [72]. 

Genipin is widely used for tissue engineering applications due to its low toxicity and negligible immunogenicity [73]. Genipin’s cytotoxicity appears to be highly dose dependent but time independent. To eliminate the toxic effect and undesired immunogenic reaction, a 0.5 mM concentration of genipin is recommended for most tissue engineering application [74]. Further dose optimization may still be required for specific applications. Table 1, elaborates on the studies that use genipin crosslinking in developing hydrogels for corneal tissue engineering [75,76,77].

#### 2.3.4. Ethyl-3-[3-dimethylaminopropyl] Carbodiimide Hydrochloride (EDC) and *N*-hydroxy-succinimide (NHS)

EDC, a zero-length crosslinking agent, commonly conjugates carboxyl or phosphate groups to primary amines through a covalent linkage. EDC forms an active O-urea that subsequently couples with the amino groups through an amide bridging [78]. As a result, a water soluble sub-product, iso-urea, is formed that can be easily eliminated by washing. This crosslinking mechanism is significantly pH dependent and the reactivity is higher with improved efficiency in an acidic environment (in presence of 2-(*N*-morpholino)ethanesulfonic acid (MES) buffer solution) compared to alkaline environments [79]. Conjugation of water soluble NHS, or its analog sulfo-NHS, in EDC enhances the efficiency of the crosslinking reaction as well as its stability. NHS esters are formed due to direct coupling between EDC/NHS and carboxyls. These NHS esters are more stable than the O-acyl-isourea intermediates and positively assist in proficient coupling of primary amines at physiological pH [80]. When using EDC for crosslinking, unreacted free groups of EDC do not remain in the material. This consequently means that the final product is not affected by EDC toxicity [81]. EDC mediated crosslinking generally utilizes cell reactive carboxylate anions (on glutamate or aspartate residues) or primary amino groups (on lysine residues), which are the major cell binding motif sites of many biomaterials for forming hydrogels, such as collagen or silk fibroin. As a result, there is a shortage of available cell binding motif sites after crosslinking [82]. To address this issue, more investigation is required to optimize the concentration of EDC for fabricating hydrogels with improved cellular reactivity, without altering surface chemistry or biomechanics. A significant improvement in mechanical integrity and stability along with cellular biocompatibility has been recorded for collagen-based scaffolds when the concentration of EDC was significantly reduced [83]. The standard concentration (100%) of carbodiimide is 11.5 mg/ml and was diluted progressively down to 0.1%. This reduction resulted in an almost 4-fold increment in the amount of free amine groups without altering the mechanics or stability in water of the resultant scaffolds. This 10-fold reduction in carbodiimide crosslinking demonstrated in near native-like cell attachment to collagen scaffolds.

From analyzing Table 1 it is found that EDC/NHS has been commonly used as a crosslinker for developing hydrogels for corneal regeneration [41,59,84,85,86,87,88,89,90]. There are considerable variations in the concentration of EDC/ NHS used in the reported articles. Almost all reported articles (8 out of 10) performed in vivo evaluations of EDC/NHS crosslinked hydrogels supported by in vitro studies using corneal specific cells.

### 2.4. Other Approaches

Several alternative crosslinking chemicals and approaches have been evaluated to improve the mechanical properties and stability of hydrogels for corneal tissue engineering [85,86,91,92,93,94,95,96,97,98,99,100,101] (Table 1). These hydrogels are made using a variety of biomaterials. For example, several studies have used novel crosslinkers on collagen hydrogels. Generation 2 polypropylenimine octaamine dendrimers have successfully crosslinked collagen hydrogels with high degree of transparency and good mechanical properties for corneal regeneration [91]. The biocompatibility of these hydrogels, in respect of cellular adhesion and proliferation, was evaluated with human corneal epithelial cells and the crosslinker showed no toxicity [91].

New crosslinkers have also been used to crosslink collagen with other biomaterials. A hybrid cross-linking system was developed using a long-range bi-functional cross-linker PEG-dibutyraldehyde (PEG-DBA) and short-range amide-type cross-linkers (EDC and NHS) to crosslink collagen–chitosan composite hydrogels [85]. The hydrogels exhibited excellent optical clarity (superior to human eye bank corneas), suturability, permeability to albumin and glucose, and significantly higher mechanical strength and elasticity, an increase of 100% and 20%, respectively, when compared to its non-hybrid counterpart. These hydrogels showed excellent biocompatibility both in vitro, using dorsal root ganglia (DRG) and human corneal epithelial cells, and in vivo using rat subcutaneous and pig cornea implants. The hydrogels supported host–graft integration with successful regeneration of corneal stroma, nerve and epithelium after 12 months implantation in pigs. 

A bio-interactive collagen-phospholipid corneal substitute was developed from interpenetrating polymeric networks, utilizing EDC/NHS crosslinked porcine atelocollagen, and PEG-diacrylate crosslinked 2-methacryloyloxyethyl phosphorylcholine (MPC) [86]. Fabricated hydrogels showed increased mechanical strength along with enhanced stability against collagenase and UV degradation and improved in vitro biocompatibility with DRG and human corneal epithelial cells. A 12 months in vivo study of hydrogels into mini pig demonstrated regeneration ability of corneal stroma, epithelium, tear film and sensory nerves. 

Chitosan and chitosan composites are also commonly used biomaterials for corneal tissue engineering. In addition to using standard crosslinking chemicals like GA, several different approaches have been explored to crosslinking chitosan. For example, an in situ formed biodegradable hydrogel was fabricated involving the water-soluble derivative of chitosan, hydroxypropyl chitosan, and sodium alginate dialdehyde for corneal endothelial regeneration [96]. Periodate oxidized alginate rapidly cross-links hydroxypropyl chitosan due to the formation of Schiff’s base between the available amino groups and aldehyde. The fabricated hydrogels were biocompatible with corneal endothelial cells, biodegradable and were evaluated as a potential scaffolds for in vivo endothelium regeneration in New Zealand rabbits.

A new post crosslinking mechanism via epoxy–amine chemistry was introduced to fabricate ultrathin (thickness in hydrated condition 50µm) chitosan–PEG hydrogel for corneal tissue regeneration [98]. The resultant hydrogel showed desirable optical transparency, biodegradability, comparable mechanical property with cornea to support a suitable mechano-responsive environment for corneal endothelial cell and supported adhesion and proliferation of sheep’s corneal endothelial cells. Ex vivo trials on ovine eyes showed that the hydrogels exhibited excellent properties for physical manipulation and implantation, which made them a potential scaffold for minimally invasive surgical procedures, such as Descemet’s Stripping Endothelial Keratoplasty (DSEK). 

Porous chitosan hydrogel sheets were developed and evaluated as a potential ophthalmic delivery substrate for levofloxacin [100]. Hydrogel sheets were fabricated spontaneously under mild conditions when a 4-arm polyethylene glycol crosslinker was mixed with aldehyde end groups (4-arm PEGCHO) and glycol chitosan (GC) at various ratios. Upon decreasing the concentration of 4-arm PEGCHO and GC, the swelling ratio of fabricated hydrogels was increased. Biocompatibility assays reported that the hydrogels were non-toxic and exhibited an excellent cytocompatibility with L929 cells. 

In addition to collagen and chitosan, there has also been considerable interest in the development of PEG based hydrogels for corneal engineering. A two-step sequential network formation approach was employed to fabricate interpenetrating hydrogels by using poly(2-hydroxyethyl methacrylate) and triethylene glycol dimethacrylate (1% *v*/*v*) as a crosslinker inside collagen immobilized PEG hydrogels [97]. Fabricated hydrogels were non-toxic and supported adhesion and proliferation of corneal epithelial cells. 

UV-initiated free radical polymerization, using a two-step sequential network formation technique was used to fabricate PEG/poly(acrylic acid) (PEG/PAA) hydrogels for corneal tissue engineering [92]. Both in vitro (using primary corneal epithelial and fibroblast cells) [92] and preliminary in vivo (New Zealand Red rabbits) [93] studies were carried out to assess the biocompatibility of the fabricated hydrogel. A similar UV-initiated free radical polymerization and crosslinking network technique was used to fabricate collagen-coupled PEG/PAA hydrogels that support corneal epithelial wound healing [94]. The bioactive surface of the hydrogels showed promising results for epithelial wound closure. In vivo results conducted on rabbits demonstrated that the implanted hydrogel supported the migration of corneal epithelial cells, although the morphology and migration rate of cells were different from normal.

A mechanically and structurally efficient artificial cornea using poly(2-hydroxyethyl methacrylate) was fabricated involving a T-style design of a keratoprosthetics [99]. *N*,*N′*-methylenebis (acrylamide) (0.5 %) was used as a crosslinker. The porous skirt was altered with hyaluronic acid and cationized gelatin, and the bottom of the optical column was coated with poly(ethylene glycol). In vitro (rabbit corneal stromal cells) and in vivo (New Zealand rabbits) analysis demonstrated that the artificial cornea was a potential corneal substitute and could be suitable for patients with corneal opacity and massive limbal stem cell deficiency. 

A saturated neoglycopolymer was developed by tandem ring opening metathesis polymerization-hydrogenation of carbohydrate-functionalized norbornenes and examined as a promising crosslinking agent for corneal tissue engineering [95]. The resultant neoglycopolymer hydrogels were superior with respect to stability, enzymatic resistivity and permeability, compared to clinically tested control materials (recombinant human collagen type III (RHC III) crosslinked using EDC/NHS) as well as demonstrating biocompatibility in vitro with human corneal epithelial cells. 

Recently a transparent, highly biocompatible, cost-effective, bio-adhesive hydrogel, using gelatin methacryloyl (GelMA) prepolymer with ∼80% methacryloyl functionalization degree (GelCORE) were fabricated utilizing photo-crosslinking with visible light (450 to 550 nm) for 60 seconds [101]. The physical properties of fabricated hydrogel can be finely adjusted by altering the photo crosslinking time and concentration of pre-polymer. In situ photo-polymerization of GelCORE improved the adhesion between the hydrogel and tissue. In vitro and in vivo (rabbit model) evaluation demonstrated that the bio-adhesive hydrogel is highly biocompatible with corneal fibroblast, efficiently enclosing stromal defects in rabbit and promoting stromal regeneration and re-epithelialization. 

### 2.5. Comparative Studies 

A number of studies have compared different crosslinking reagents (Table 2) and their effectiveness at modifying physical properties, chemical structure, mechanical characteristics and biological effects [41,59,60,91,102,103].

In one study, collagen solutions (2–4%) were crosslinked with EDC, GA or polypropyleneimine-octa-amine dendrimers [91]. The multi-functional dendrimers were introduced after the activation of the carboxylic acid groups of glutamic and aspartic acid residues in collagen. The dendrimer crosslinked collagen hydrogels exhibited significantly higher optical transparency than EDC and GA crosslinked hydrogels as well as higher glucose permeability compared to human corneas. Adhesion and proliferation of human corneal epithelial cells were supported by dendrimer crosslinked collagen hydrogels without inducing cellular toxicity. 

The crosslinking kinetics and properties of recombinant human atelocollagen type III hydrogels were examined using two different crosslinking agents: (i) sterically bulky carbodiimide, *N*-cyclohexyl-*N′*-(2-morpholinethyl) carbodiimide metho-p-toluenesulfonate (CMC) and (ii) EDC [103]. The major advantage of CMC crosslinking was that it supported crosslinking at room temperature (25 °C), while EDC crosslinking process was too fast to control at room temperature. Therefore, it is required to be executed at lower temperatures. CMC crosslinked hydrogels were significantly stiffer and exhibited higher collagenase resistivity compared to EDC crosslinked hydrogels. Comparable biocompatibility, in vitro (human corneal epithelial and endothelial cells, DRGs from chick embryos) and in vivo (mouse model), was demonstrated for both crosslinked hydrogels [103].

Another comparative analysis was conducted between EDC and GA to identify the more appropriate crosslinker for hyaluronic acid (HA) hydrogels. These hydrogels were developed as cell delivery vehicles for corneal endothelial cell therapy [59]. Water uptake capacity and enzymatic degradability were significantly decreased for HA hydrogels crosslinked with GA. EDC crosslinked HA hydrogels had a faster degradation rate and smoother surfaces. Lower cytotoxicity for the corneal endothelial cell was also recorded for EDC crosslinked HA hydrogels compared with GA crosslinked HA hydrogels. This study identified EDC as a better option for HA crosslinking. Comparative in vivo evaluation in rabbits for 24 weeks was also carried out to examine the ocular biocompatibility of the HA hydrogels crosslinked with EDC and GA [60]. EDC crosslinked HA hydrogels supported better ocular biocompatibility than GA crosslinked HA hydrogels. No significant inflammatory cell infiltration or foreign body reaction was observed after implantation for non-cross-linked or EDC cross-linked HA hydrogels, whereas adverse inflammatory reaction was quite prominent for GA crosslinked HA hydrogel. 

Similar comparative studies between EDC and GA were carried out using gelatin [41]. In vitro analysis using primary rat iris pigment epithelial cells demonstrated that the cells cultured on EDC crosslinked gelatin hydrogels showed lower lactate dehydrogenase activity, cytotoxicity, and interleukin-1β and tumor necrosis factor-α levels compared to cells cultured on GA cross-linked gelatin hydrogels. In vivo analysis in rabbit model also reported better biocompatibility, less toxicity and fewer adverse effects for EDC cross-linked gelatin hydrogels compared to GA. 

In vivo ocular biocompatibility of genipin and GA crosslinked chitosan hydrogel were compared in rabbits [102]. Genipin crosslinked implanted hydrogels showed no signs of ocular inflammation in the anterior chamber of the eye, enhanced the preservation of corneal endothelial cell density as well as supported better anti-inflammatory activities, when compared with non crosslinked and GA-crosslinked chitosan hydrogels.

## 3. Crosslinking Strategies for Injectable Hydrogel

**Figure 3:**
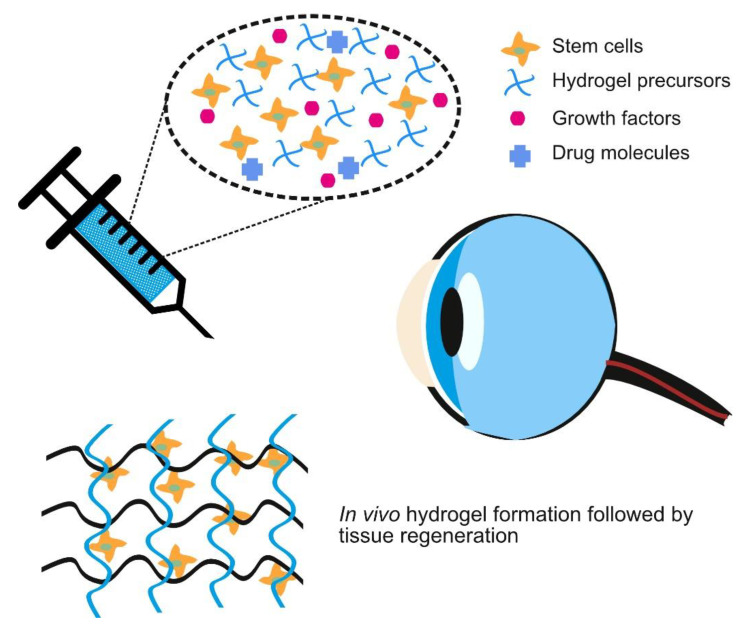
Schematic representing the potential different components of an injectable hydrogel to be used for corneal repair.

Injectable hydrogels are hydrogels that form after injection into the body and have been used for drug delivery, tissue defects repair and as cell delivery vehicles [104]. In the eye, injectable hydrogels have been examined as a substitute for vitreous humor and more recently, for corneal defect repair [104]. Figure 3 represents such an idealized future situation where hydrogel precursors, loaded with stem cells and bioactive molecules, can be injected and form a hydrogel with desirable characteristics in vivo. The composition and quantity of bioactive molecules could be easily varied depending on the clinical requirements and could even be made patient specific. Injectable hydrogels demonstrate more potential than pre-formed hydrogels for the delivery of a therapeutic payload [105,106,107]. The most important properties that affect swelling, drug release rate and oxygen permeability of the hydrogels are the molecular weight of a polymer between two crosslink points and the mesh size. The gelation, crosslinking and the application of injectable hydrogels for corneal regeneration will be discussed here.

### 3.1. Gelation and Formulation

Injectable hydrogels are composed of synthetic or naturally derived hydrophilic polymers that are able to crosslink in situ following a variety of mechanisms [108]. The Food and Drug Administration, USA (FDA) has approved several synthetic polymers including polyvinyl alcohol (PVA), PEG, PAA, poly(*N*-isoproylacrylamine) (PNIPAAm) and Pluronic F-127. These polymers are able to crosslink hydrophilic co-polymers or homo-polymers and effectively develop block co-polymers with other polymers [104]. Several naturally derived polymers such as polysaccharides (alginate, chitosan, hyaluronic acid and dextran) and proteins (collagen and gelatin) have also been used to develop injectable hydrogels for ophthalmic applications [104]. These hydrogels use covalent crosslinking, the Diels–Alder reaction, enzyme reactions to effect in situ Michael addition, Schiff base formation and click chemistry to form stable hydrogels [104]. Crosslinking of injectable hydrogels is often initiated by altering physico-chemical parameters such as temperature, pH, ionic strength, the glucose concentration or mechanical stress. These physico-chemical parameters induce phase separation and structural alteration of polymer chains to develop a crosslinked network [109]. Stimuli responsive polymers such as thermo-responsive PEG, PNIPAAm and Pluronic F-127 or pH-responsive polyacrylic acid (PAAc) and chitosan, can be easily formed by crosslinking an injected hydrogel network [104]. 

### 3.2. The Injectable Hydrogels in Treatment

In situ forming hydrogels are an efficient way to repair corneal wounds and defects by delivering drugs and cells to the damaged region of the cornea [110]. To examine the effectiveness of induced pluripotent stem cells (iPSCs) in bioengineered cornea for corneal regeneration, a thermo-responsive injectable amphiphatic carboxymethylhexanoyl chitosan (CHC) nanoscale hydrogel was synthesized [111]. This hydrogel supported increased cell viability and gene expression associated with stem cells. In vivo experiments involved administrating the injectable iPSCs laden CHC hydrogel in a defect site of the rat cornea. CHC hydrogels improved regeneration of damaged cornea by down regulating oxidative stress that led to the restoration of the corneal epithelial thickness. Therefore, CHC hydrogels are a potential scaffold for stem cell delivery to improve corneal wound healing [111].

A PEG based injectable hydrogel was developed by incorporating Tyr-Arg-Gly-AspSer (YRGDS) peptides [112]. Keratocytes encapsulated in these hydrogels maintained their viability over 4 weeks and displayed genetic and morphological characteristics associated with healthy, functional keratocytes. However, further advancement in the development of PEG based hydrogels as a cell based therapeutic approach for keratoconus treatment is required as it failed to restore the keratocyte phenotype completely.

A novel LiQD cornea has recently been developed as an alternative to donated corneas for transplantation [113]. This cell-free hydrogel liquid was synthesized using short collagen-like peptides combined with PEG and blended with fibrinogen. In vitro and in vivo analysis demonstrated that this self-assembled LiQD cornea is biocompatible, non-toxic and significantly reduced the risk of immune rejection associated with xenogeneic materials. LiQD cornea is also capable of undergoing rapid in situ gelation and may serve as a potential material for corneal regeneration.

An in situ, rapidly formed, PEG-based doxycycline laden transparent hydrogel was successfully fabricated through thiol reactions and was examined for corneal wound healing applications [114]. This hydrogel exhibited a prolonged release of doxycycline (up to 7 days) and was able to resist the structural deformation under shearing force. Remarkably, a decline in the production of matrix metalloproteinase-9 (MMP-9) was shown through immunofluorescence and histology analysis. This result supported better corneal healing for these hydrogels. Thus, PEG-based homo-polymers and co-polymers are an attractive choice for corneal repair.

An injectable hydrogel utilizing the thermo-responsive co-polymer of poly(lactic-co-glycolic acid) (PLGA) and PEG was synthesized through sol-gel transition at temperatures ranging between 5 and 60 °C [115]. In vitro biocompatibility tests showed that these hydrogels supported proliferation and migration of epithelial cells. In vivo (rabbit model) results of implanted hydrogels demonstrated that keratocytes retain a natural morphology appearance and there was desirable healing of corneal wounds [115].

There is limited availability of FDA approved injectable hydrogels for commercial use and they have only been used to prompt the healing process post ocular surgery. Ongoing investigations are aiming to use injectable hydrogels as a delivery vehicle of drugs and cells for corneal regeneration. They are also trying to overcome the complications associated with stem cell research. It may take several years before injectable hydrogels are clinically available as a delivery vehicle of cells and drugs for corneal repair and regeneration.

## 4. Impact of Crosslinkers on Hydrogel Characteristics

**Figure 4:**
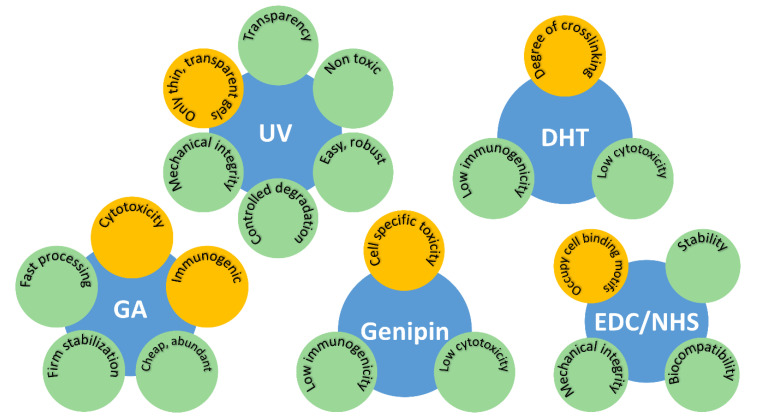
Schematic representation summarizing the pros (green) and cons (orange) of commonly used crosslinking techniques and chemical additives in terms of their impact on hydrogel characteristics.

The type and duration of crosslinking affects the physical properties and biological compatibility of many hydrogels. For example, an increase in crosslinking will increase degradation resistance for most hydrogels. Similarly, hydrogel formation may often need to take place with cells and/or proteins present, thus necessitating the crosslinking process to be cytocompatibility. Thus, it is desirable to control the crosslinking process. An overview of the effect of different crosslinking actions on hydrogel properties is shown below (Figure 4).

### 4.1. Mechanical Characteristics

The mechanical characteristics of hydrogels are dependent on the type and magnitude of crosslinking that has been applied to them. In general, the use of chemical crosslinking reagents results in stable hydrogels with better mechanical properties while other methods such as photo-crosslinking can provide better cytocompatibility since no additional chemical agents are required. The mechanical properties of hydrogels are often described as viscoelastic, where they exhibit both viscous and elastic characteristics [116,117]. This results in time dependent deformation behaviors such as creep, relaxation and time dependent recovery. The viscoelastic characteristics of hydrogels tend to be dependent on the degree and density of crosslinking. Alternatively, many studies describe the behavior of hydrogels as being elastic with similar properties to rubber. The correct description of the hydrogel mechanical characteristics depends on the material composition and type of crosslinking. The crosslinks that improve mechanical strength also increase viscosity, reduces solubility and reduce glass transition temperature (T_g_).

At the macro-scale, the mechanical properties of hydrogels affect their stability, strength and stiffness while at the micro-scale mechanical properties can affect how the hydrogels interact with cells and affect cell signaling, proliferation, migration, and differentiation [118,119]. Hydrogel stiffness can be adjusted by tuning the crosslinker density, crosslinking time and the type of precursors used [120].

Both UV and DHT initiated crosslinking have been shown to improve the tensile strength of collagen based hydrogels although they led to fragmentation of the collagens basic structure [39,44]. Another limitation with photo-crosslinking is the inability of light to penetrate deep into a material, although this is less of a problem for thin, transparent hydrogels [54]. Photo-crosslinking can be used to finely control mechanical properties of hydrogels by adjusting the time of exposure and light intensity [101].

2 polypropylenimine octaamine dendrimers have been shown to produce transparent collagen hydrogels with good mechanical properties [91]. Interestingly, a hybrid approach of using a long-range bi-functional crosslinker (PEG-DBA) and short-range amide-type crosslinkers (EDC and NHS) was able to produce a collagen-chitosan hydrogel that was transparent, had good mechanical properties and could be sutured [85].

### 4.2. Degradation and Structural Properties

Chemical crosslinking produces covalently bonded hydrogels. These covalent bonds between the polymeric chains can be broken down by photo-catalytic cleaving, ester or enzymatic hydrolysis [121]. To provide adequate support as a scaffold, the hydrogels should degrade at a rate that matches new tissue formation so there is no loss of strength or function. To do this, chemical crosslinking parameters can be tailored as required by varying crosslinking time, crosslinker concentration and precursors [122]. Interestingly, degradation, which is a chemical process, alters the physical surroundings of cells and in turn can affect how those cells behave [123]. 

Hydrogel mesh size impacts solute transmission through the structure [124]. Particles that are larger than the effective pore size in the mesh will thus be excluded. A hydrogel with an asymmetric mesh size can provide bio-separation, that is a high solute flux, as well as selective cell capture and encapsulation [125]. Mesh size can also affect the hydrogel degradation rate. Hydrogels with a high crosslinking density, generally achievable through the use of chemical crosslinkers, reduces mesh size and slows down degradation [126]. The reduced mesh size also slows down the transport of larger molecules, like enzymes, thus limiting access of the enzymes to degradation sites [121]. However, the reduction of molecular diffusion due to smaller mesh size can pose a problem for nutrients transfer, which may be required for the survival of encapsulated cells.

Aimetti et al. [127] developed a hydrogel that degrades via surface erosion. A human neutrophil elastase (HNE) sensitive peptide was used as a crosslinker in PEG hydrogels, via thiol–ene photopolymerization. The high crosslinking density resulted in a reduced mesh structure, limiting HNE diffusion into the hydrogel. Thus, degradation gradually occurred via surface erosion and through this process, a protein entrapped physically in the hydrogel was released. 

To mimic natural, soft tissues and their multi-scale, hierarchical structure, an ideal hydrogel for tissue engineering would need to be anisotropic and have a highly ordered architecture [128]. Hydrogel structural hierarchy is an important consideration in designing innovative hydrogels [129]. While physical methods such as plastic compress [130,131] and magnetic fields [132,133] can be used to organize the structure of some hydrogels, controlled crosslinking procedures may be adopted to develop hydrogels with specific architectures. For example, interpenetrating polymer network hydrogels, allow hydrogels to be formed that contains a desirable structural hierarchy by varying the ratio of the different polymers [134].

In addition to affecting the hydrogels mechanical characteristics, UV initiated crosslinking can also improve the degradation resistance of collagen hydrogels to different enzymes [46]. UV crosslinking of GelMA hydrogels has been shown to reduce the rate of degradation [53]. Crosslinking gelatin using DHT has also been able to achieve controlled release of hydrogel embedded bioactive molecules [37]. Use of GA also increases degradation resistance of gelatin [55]. Similarly, combined use of EDC and NHS produced crosslinked atelocollagen hydrogels that were stable against collagenase and UV degradation [86].

### 4.3. Toxicity and Biocompatibility

Crosslinkers have an important role in modulating chemical and mechanical features of hydrogels so that they lead to a desirable cell response [135]. Changes in the hydrogel properties after crosslinking can affect the behavior and activity of cells in contact with the material. Ideally, a crosslinker must be able to improve mechanical properties while maintaining the biocompatibility of the scaffold and without generating any toxic bi-product [136].

UV crosslinking is not only able to produce a biocompatible hydrogel with no toxic residuals [53], but UV irradiation has also been safely used on the cornea and posterior segment of the eye [52]. However, the wavelength and intensity of UV light need to be considered as over-exposure to UV or too low a wavelength can result in apoptosis. 

In spite of its toxicity, GA is one of the mostly used crosslinkers that has been studied by many research groups for tissue engineering application [137]. Many studies reported that GA leaching undergoes simultaneously with scaffold degradation and results in cytotoxicity. Hence, GA residues can be harmful to the cells over long periods [138]. In contrast, genipin is less cytotoxic and still maintains a strong crosslinking ability. Safety issues regarding the use of genipin are still a concern as cellular behavior has been shown to vary significantly after crosslinking depending on the cell type [102]. Genipin can promote the differentiation of neurite cells and accelerate dose dependent neurite outgrowth [139]. However, immediate apoptosis was demonstrated with liver and dermal cells after genipin crosslinking. The concentration of genipin should be optimized for tissue specific studies using different cell types [135]. 

An alternative to GA and genipin crosslinking, EDC/NHS efficiently crosslinks amino acid based biomaterials with favorable cellular performance [90]. EDC/NHS results in fewer crosslinks compared to GA but it does not present itself in the final product, thus reducing the potential for toxicity [135,140]. However, EDC/NHS crosslinking occupies integrin binding sites in the same carboxylic chain of biopolymers, which are pivotal for integrin-mediated cell interactions [80]. Therefore, there is a requirement to try to conserve the active cell binding sites during crosslinking without altering the chemistry of biomaterial. The use of EDC-NHS creates stable and mechanically strong hydrogels with collagen [83]. An EDC concentration of 0.1% for collagen is recommended to maintain biocompatibility while still improving strength. When applied to hyaluronic acid hydrogels EDC led to lower cytotoxicity but faster degradation and a reduced water uptake compared to GA [59].

## 5. Challenges and Future Perspective

There are several challenges that need to be addressed before crosslinked hydrogels and scaffolds are more commonly used on patients for corneal tissue engineering. Sterilization of the biomaterials can cause some difficulties since most natural and synthetic polymers undergo degradation during radiation, heat or chemical sterilization processes [141]. These issues are made worse when bioactive molecules, proteins and drugs are incorporated into the polymer matrix [142]. In addition, some hydrogel polymers have a specific shelf life after which there is a reduced ability to form crosslinks. Chemical crosslinking agents support the fabrication of hydrogels with well-defined characteristics that may hinder the availability and stability of biopharmaceuticals. Physical crosslinking may be better at conserving the stability of incorporated biopharmaceuticals, but it is more difficult to control the release of drugs and the degradation kinetics of these hydrogels. 

Optimization of crosslinking is required to successfully control the release of drugs or bioactive molecules from hydrogels. For drugs that are chemically bound to the hydrogel, these can be released via degradation. However, controlling the degradation kinetics after encapsulating drug molecules is challenging. The degradation rate of drug loaded hydrogels and their drug release profiles may vary from patient to patient depending on many factors including age, sex and health of the patients [141]. Rapid degradation of the hydrogel may accelerate the release of drugs, while inhibiting degradation may lead to incomplete drug release.

Another challenge is how best to control the spatial distribution of cells throughout a hydrogel. One approach to doing this is to employ 3D bioprinting to engineer constructs with a high degree of precision. Most hydrogels used for 3D bioprinting (called bioinks) utilize UVA light to generate crosslinks and form a stable hydrogel. This technology has the potential to assist clinicians and researchers to produce innovative constructs to address corneal donor shortages. In addition, 3D bioprinting could be used to incorporate drugs or biological reagents into the hydrogels. Recently a number of groups have started exploring the application of 3D bioprinting for corneal tissue engineering [143,144,145,146].

## 6. Conclusions

This review has explored different crosslinking mechanisms that are used to fabricate hydrogels for corneal regeneration. While there is not one ideal crosslinker with all the desirable properties for fabricating hydrogels for corneal regeneration, many of the crosslinking techniques described here are beneficial in controlling the mechanical behavior of hydrogels without adversely affecting their biocompatibility. Overall, variations in the type of hydrogel material selected and the crosslinking dosage significantly affect the properties of the resultant scaffold. Detailed studies that focus on the optimization of crosslinker concentration, cytocompatibility and biocompatibility are necessary to understand the advantages and limitations of each crosslinking approach. 

## Figures and Tables

**Table 1:** Complete details of the different crosslinkers used in corneal regeneration to synthesize corneal hydrogels identified in this review.

Paper	Biomaterial	Crosslinkers	Fabrication Method	Cell Study	In Vivo Study
Glutaraldehyde (GA)					
[57]	Gelatin	10% GA at 4 °C for 14 h	Lyophilization	-	Pigmented rabbits
[58]	Collagen I + chondroitin sulphate	GA conc. (0.02, 0.04, 0.06 and 0.08%)	Air-lifted and maintained at air-liquid interfaces	Keratocytes ± corneal epithelial and endothelial cells	-
[67]	Collagen + poly(ethylene oxide dialdehyde)	GA	Air drying and argon plasma surface modification	Human epithelial cells	-
[59]	Hyaluronic acid	100 mM GA at 25 °C for 2 days	Solution casting and air-drying	Corneal endothelial cells	-
[60]	Hyaluronic acid	100 mM GA at 25 °C for 2 days	Solution casting and air-drying	-	New Zealand white rabbits
[41]	Gelatin	50 mM GA at 25 °C for 80 min	Solution casting and air-drying	Rat iris pigment epithelial cells	New Zealand white rabbits
[61]	Collagen, copolymers of collagen and TERP	0.22% GA at room temperature for 7 days	Air drying	-	Adult laboratory beagles
[62]	Amniotic membrane	0.1% GA and hyperdried	Far infrared rays and microwaves	-	Three eyes of three patients
[63]	Hyaluronic acid + itaconic acid + PEGDE	GA under acidic pH	Air drying	Human corneal epithelial cell line	New Zealand white rabbits
[64]	Amniotic membrane (AM)	0.05 mmol GA per mg AM	Air drying	Limbal epithelial cells	-
[65]	Canine AM + atelocollagen	0.1% GA	Air drying	Canine corneal epithelial cells	-
[66]	Carboxymethyl chitosan + poloxamer	1% GA for 1 h at 50 °C	Air drying	Human corneal epithelial cells	-
**1,4-Butanediol diglycidyl ether (BDDGE)**					
[69]	Chitosan + gelatin + chondroitin sulfate	0.5% BDDGE	Lyophilization	Human and rabbit keratocytes	-
[70]	Porcine collagen type I	BDDGE at pH 11	Air drying	Human corneal epithelial and rodent DRG cell	-
**Genipin (GP)**					
[75]	Chitosan + collagen, cellulose or elastin	GP (40 µL)	Air drying	Human corneal epithelial cells	-
[76]	Chitosan	0.5–5.0 mM GP	Lyophilization	Human corneal epithelial cells	-
[77]	Carboxymethyl chitosan + poloxamer	02–0.8% GP	Lyophilization	-	New Zealand rabbits (ex vivo)
**Ethyl-3-[3-dimethylaminopropyl] carbodiimide hydrochloride (EDC) & N-hydroxy-succinimide (NHS)**					
[87]	Amniotic membranes (AM)	0–0.25 mmol EDC per mg AM EDC:NHS molar ratios = 5:1	Immersion	Limbal epithelial cells	New Zealand white rabbits
[41]	Gelatin	50 mM EDC	Solution casting and air-drying	Rat iris pigment epithelial cells	New Zealand white rabbits
[88]	Hyaluronic acid	10 mM EDC at 25 °C for 2 days	Lyophilization	Corneal endothelia	New Zealand white rabbits
[89]	Hyaluronic acid	10 mM EDC	Lyophilization	Corneal endothelia	New Zealand white rabbits
[90]	Collagen I + gelatin (Col/Gel)	EDC:NHS:(Col/Gel) = 1:1:12 for 4 h	Lyophilization	Human mesenchymal stem cells	-
**Other crosslinkers**					
[91]	Type I collagen	Generation 2 polypropyleneimine octaamine dendrimers	Chemical crosslinking	Human corneal epithelial cells	-
[92,93]	PEG and PAAc double network hydrogel	50% acrylic acid 1% *v/v* with respect to hydroxyl-2-methyl propiophenone and triethylene glycol dimethacrylate	Two-step sequential network formation technique	Primary corneal epithelial and fibroblast cells	New Zealand Red rabbits
[94]	Collagen coupled PEG/PAAc	1% triethylene glycol dimethacrylate for 24 h at room temperature	UV- free radical polymerization	Rabbit corneal cell line	New Zealand Red rabbits
[85]	PEG-stabilized collagen + chitosan	Hybrid cross-linking system comprising of a long-range bi-functional cross-linker	Chemical crosslinking	Human corneal epithelial cells, and DRG	Yucatan porcine cornea and rat subcutaneous
[86]	Collagen–phosphorylcholine	PEG diacrylate initiated by ammonium persulphate or 0.5% Irgacure 2959	Photopolymerization	Human corneal epithelial cell line and DRG	Mini-pigs and New Zealand white rabbits
[95]	Neoglycopolymer—recombinant collagen III	Carbohydrate-functionalized norbornenes	Tandem ring-open metathesis polymerization hydrogenation	Human corneal epithelial cells	-
[96]	Hydroxypropyl chitosan (HPCTS)	Sodium alginate dialdehyde (20 mg/mL) mixed equal volume with HPCTS	Self-cross-linking process of chitosan and oxidized alginate	Corneal endothelial cells	New Zealand rabbits
[97]	Collagen I-Immobilized PEG	1% Triethylene glycol dimethacrylate and poly(2-hydroxyethyl methacrylate)	UV-initiated free radical polymerization	Human corneal epithelial cells	-
[98]	Chitosan + PEG	Diepoxy-PEG:cystamine (4:1 molar ratio)	Casting and chemical crosslinking for 24 h at 25 °C	Sheep endothelial cell	Ovine eyes (ex vivo)
[99]	Poly(2-hydroxyethyl methacrylate)	N, N′-methylenebis 0.5% acrylamide	Polymerization and molding processes	Rabbit corneal stromal cells	New Zealand rabbits
[100]	Levofloxacin loaded glycol chitosan	4-arm polyethylene glycol with aldehyde end groups (4-arm PEG-CHO)	Chemical crosslinking	L-929 cells	-
[101]	GelCORE bioadhesive hydrogels	Photocrosslinking with visible light (450 to 550 nm)	Lyophilization, chemical and photo-crosslinking	Corneal fibroblast cells	New Zealand white rabbits

**Table 2:** Works identified in this review focusing on the comparison of different crosslinkers used to develop corneal hydrogels.

Paper	Biomaterial	Crosslinkers & Concentration	Results
[91]	Type I collagen	Generation 2 polypropyleneimine octaamine dendrimers: EDC: molar ratio 1:1 GA: 0.02%.	Dendrimer-crosslinked gel had no cellular toxicity and higher glucose permeability than natural human cornea and more transparent than GA/EDC crosslinked gels
[59]	Hyaluronic acid (HA)	EDC:100 mM GA:100 mM	EDC-HA was more transparent, smoother surface, faster degradation and lower toxicity than GA-HA
[60]	Hyaluronic acid (HA)	EDC:100 mM GA:100 mM	EDC-HA gel had no adverse inflammatory reaction GA-HA gel induced significant inflammatory cell infiltration and foreign body reaction observed
[41]	Gelatin	EDC:50 mM GA:50 mM	EDC-gelatin was biocompatible without causing toxicity GA-gelatin showed significant inflammatory reaction
[102]	Chitosan	10 mM GA 10 mM Genipin (GP)	GP crosslinked implants were more biocompatible without providing significant intraocular inflammation
[103]	Recombinant human atelocollagen type III	EDC: 0.3 ME (Molar equivalent) CMC: 2.0 ME.	CMC crosslinked samples had comparable properties to EDC crosslinked hydrogels

## Data Availability

The data presented in this study are available on request from the corresponding author.

## References

[1] Whitcher J.P., Srinivasan M., Upadhyay M.P. (2001). Corneal blindness: A global perspective. Bull. World Health Organ..

[2] Thompson R.W., Price M.O., Bowers P.J., Price F.W. (2003). Long-term graft survival after penetrating keratoplasty. Ophthalmology.

[3] Shimazaki J., Shinozaki N., Shimmura S., Holland E.J., Tsubota K. (2004). Efficacy and Safety of International Donor Sharing: A Single-Center, Case-Controlled Study on Corneal Transplantation. Transplantation.

[4] Cao K.Y., Dorrepaal S.J., Seamone C., Slomovic A.R. (2006). Demographics of corneal transplantation in Canada in 2004. Can. J. Ophthalmol..

[5] Salvador-Culla B., Kolovou P.E. (2016). Keratoprosthesis: A Review of Recent Advances in the Field. J. Funct. Biomater..

[6] Dua H.S., Gomes J.A., King A.J., Maharajan V. (2004). The amniotic membrane in ophthalmology. Surv. Ophthalmol..

[7] Shimazaki J., Shinozaki N., Tsubota K. (1998). Transplantation of amniotic membrane and limbal autograft for patients with recurrent pterygium associated with symblepharon. Br. J. Ophthalmol..

[8] Tseng S.C., Espana E.M., Kawakita T., Di Pascuale M.A., Li W., He H., Liu T.-S., Cho T.-H., Gao Y.-Y., Yeh L.-K. (2004). How Does Amniotic Membrane Work?. Ocul. Surf..

[9] Connon C.J., Doutch J., Chen B., Hopkinson A., Mehta J.S., Nakamura T., Kinoshita S., Meek K.M. (2009). The variation in transparency of amniotic membrane used in ocular surface regeneration. Br. J. Ophthalmol..

[10] Shortt A.J., Secker G.A., Rajan M.S., Meligonis G., Dart J.K., Tuft S.J., Daniels J.T. (2008). Ex Vivo Expansion and Transplantation of Limbal Epithelial Stem Cells. Ophthalmology.

[11] Dua H.S., Rahman I., Miri A., Said D.G. (2010). Variations in amniotic membrane: Relevance for clinical applications. Br. J. Ophthalmol..

[12] Wilson S.L., Sidney L.E., Dunphy S.E., Rose J.B., Hopkinson A. (2013). Keeping an Eye on Decellularized Corneas: A Review of Methods, Characterization and Applications. J. Funct. Biomater..

[13] Lynch A.P., Ahearne M. (2013). Strategies for developing decellularized corneal scaffolds. Exp. Eye Res..

[14] Lynch A.P., Wilson S.L., Ahearne M. (2016). Dextran Preserves Native Corneal Structure during Decellularization. Tissue Eng. Part C Methods.

[15] Yin H., Qiu P., Wu F., Zhang W., Teng W., Qin Z., Li C., Zhou J., Fang Z., Tang Q. (2016). Construction of a Corneal Stromal Equivalent with SMILE-Derived Lenticules and Fibrin Glue. Sci. Rep..

[16] Ahearne M., Fernández-Pérez J., Masterton S., Madden P.W., Bhattacharjee P. (2020). Designing Scaffolds for Corneal Regeneration. Adv. Funct. Mater..

[17] Fernández-Pérez J., Kador K.E., Lynch A.P., Ahearne M. (2020). Characterization of extracellular matrix modified poly(ε-caprolactone) electrospun scaffolds with differing fiber orientations for corneal stroma regeneration. Mater. Sci. Eng. C.

[18] Xu W., Wang Z., Liu Y., Wang L., Jiang Z., Li T., Zhang W., Liang Y. (2018). Carboxymethyl chitosan/gelatin/hyaluronic acid blended-membranes as epithelia transplanting scaffold for corneal wound healing. Carbohydr. Polym..

[19] Wu Z., Kong B., Liu R., Sun W., Mi S. (2018). Engineering of Corneal Tissue through an Aligned PVA/Collagen Composite Nanofibrous Electrospun Scaffold. Nanomaterials.

[20] Ruberti J.W., Zieske J.D. (2008). Prelude to corneal tissue engineering—Gaining control of collagen organization. Prog. Retin. Eye Res..

[21] Zhao Y., Fan J., Bai S. (2019). Biocompatibility of injectable hydrogel from decellularized human adipose tissue in vitro and in vivo. J. Biomed. Mater. Res. Part B Appl. Biomater..

[22] Pollinger K., Hennig R., Ohlmann A., Fuchshofer R., Wenzel R., Breunig M., Tessmar J., Tamm E.R., Goepferich A. (2013). Ligand-functionalized nanoparticles target endothelial cells in retinal capillaries after systemic application. Proc. Natl. Acad. Sci. USA.

[23] Luschmann C., Tessmar J., Schoeberl S., Strauss O., Framme C., Luschmann K., Goepferich A. (2013). Developing an in situ nanosuspension: A novel approach towards the efficient administration of poorly soluble drugs at the anterior eye. Eur. J. Pharm. Sci..

[24] Lee S.S., Hughes P., Ross A.D., Robinson M.R. (2010). Biodegradable Implants for Sustained Drug Release in the Eye. Pharm. Res..

[25] Lin C.-C., Anseth K.S. (2009). PEG Hydrogels for the Controlled Release of Biomolecules in Regenerative Medicine. Pharm. Res..

[26] Vermonden T., Censi R., Hennink W.E. (2012). Hydrogels for Protein Delivery. Chem. Rev..

[27] Van Tomme S.R., Storm G., Hennink W.E. (2008). In situ gelling hydrogels for pharmaceutical and biomedical applications. Int. J. Pharm..

[28] Oryan A., Kamali A., Moshiri A., Baharvand H., Daemi H. (2018). Chemical crosslinking of biopolymeric scaffolds: Current knowledge and future directions of crosslinked engineered bone scaffolds. Int. J. Biol. Macromol..

[29] Reddy N., Reddy R., Jiang Q. (2015). Crosslinking biopolymers for biomedical applications. Trends Biotechnol..

[30] Atyabi F., Bakhshandeh H., Soleimani M., Hosseini S.S., Hashemi H., Shabani I., Shafiee A., Nejad A.H.B., Erfan M., Dinarvand R. (2011). Poly (ε-caprolactone) nanofibrous ring surrounding a polyvinyl alcohol hydrogel for the development of a biocompatible two-part artificial cornea. Int. J. Nanomed..

[31] Ahearne M., Liu K.-K., El Haj A.J., Then K.Y., Rauz S., Yang Y. (2010). Online Monitoring of the Mechanical Behavior of Collagen Hydrogels: Influence of Corneal Fibroblasts on Elastic Modulus. Tissue Eng. Part C Methods.

[32] Gostynska N., Krishnakumar G.S., Campodoni E., Panseri S., Montesi M., Sprio S., Kon E., Marcacci M., Tampieri A., Sandri M. (2017). 3D porous collagen scaffolds reinforced by glycation with ribose for tissue engineering application. Biomed. Mater..

[33] Ruini F., Tonda-Turo C., Chiono V., Ciardelli G. (2015). Chitosan membranes for tissue engineering: Comparison of different crosslinkers. Biomed. Mater..

[34] Haugh M.G., Jaasma M.J., O’Brien F.J. (2009). The effect of dehydrothermal treatment on the mechanical and structural properties of collagen-GAG scaffolds. J. Biomed. Mater. Res. Part A.

[35] Gomes S., Rodrigues G., Martins G., Henriques C., Silva J. (2013). In vitro evaluation of crosslinked electrospun fish gelatin scaffolds. Mater. Sci. Eng. C.

[36] Ratanavaraporn J., Rangkupan R., Jeeratawatchai H., Kanokpanont S., Damrongsakkul S. (2010). Influences of physical and chemical crosslinking techniques on electrospun type A and B gelatin fiber mats. Int. J. Biol. Macromol..

[37] Hori K., Sotozono C., Hamuro J., Yamasaki K., Kimura Y., Ozeki M., Tabata Y., Kinoshita S. (2007). Controlled-release of epidermal growth factor from cationized gelatin hydrogel enhances corneal epithelial wound healing. J. Control. Release.

[38] Watanabe R., Hayashi R., Kimura Y., Tanaka Y., Kageyama T., Hara S., Tabata Y., Nishida K. (2011). A Novel Gelatin Hydrogel Carrier Sheet for Corneal Endothelial Transplantation. Tissue Eng. Part A.

[39] Weadock K.S., Miller E.J., Bellincampi L.D., Zawadsky J.P., Dunn M.G. (1995). Physical crosslinking of collagen fibers: Comparison of ultraviolet irradiation and dehydrothermal treatment. J. Biomed. Mater. Res..

[40] Germain L., Auger F.A., Grandbois E., Guignard R., Giasson M., Boisjoly H., Guérin S.L. (1999). Reconstructed Human Cornea Produced in vitro by Tissue Engineering. Pathobiology.

[41] Lai J.-Y. (2010). Biocompatibility of chemically cross-linked gelatin hydrogels for ophthalmic use. J. Mater. Sci. Mater. Med..

[42] Lew D.-H., Liu P.H.-T., Orgill D.P. (2007). Optimization of UV cross-linking density for durable and nontoxic collagen GAG dermal substitute. J. Biomed. Mater. Res. Part B Appl. Biomater..

[43] Davidenko N., Bax D.V., Schuster C.F., Farndale R.W., Hamaia S.W., Best S.M., Cameron R.E. (2016). Optimisation of UV irradiation as a binding site conserving method for crosslinking collagen-based scaffolds. J. Mater. Sci. Mater. Med..

[44] Ahearne M., Yang Y., Then K.Y., Liu K.-K. (2007). Non-destructive mechanical characterisation of UVA/riboflavin crosslinked collagen hydrogels. Br. J. Ophthalmol..

[45] Ahearne M., Coyle A. (2016). Application of UVA-riboflavin crosslinking to enhance the mechanical properties of extracellular matrix derived hydrogels. J. Mech. Behav. Biomed. Mater..

[46] Heo J., Koh R.H., Shim W., Kim H.D., Yim H.-G., Hwang N.S. (2015). Riboflavin-induced photo-crosslinking of collagen hydrogel and its application in meniscus tissue engineering. Drug Deliv. Transl. Res..

[47] Ohan M.P., Dunn M.G. (2003). Glucose stabilizes collagen sterilized with gamma irradiation. J. Biomed. Mater. Res. Part A.

[48] Mi S., Khutoryanskiy V.V., Jones R.R., Zhu X., Hamley I.W., Connon C.J. (2011). Photochemical cross-linking of plastically compressed collagen gel produces an optimal scaffold for corneal tissue engineering. J. Biomed. Mater. Res. Part A.

[49] Wollensak G., Spoerl E., Seiler T. (2003). Riboflavin/ultraviolet-a–induced collagen crosslinking for the treatment of keratoconus. Am. J. Ophthalmol..

[50] Applegate M.B., Partlow B.P., Coburn J., Marelli B., Pirie C., Pineda R., Kaplan D.L., Omenetto F.G. (2016). Photocrosslink-ing of silk fibroin using riboflavin for ocular prostheses. Adv. Mater..

[51] Bhattacharjee P., Fernández-Pérez J., Ahearne M. (2019). Potential for combined delivery of riboflavin and all-trans retinoic acid, from silk fibroin for corneal bioengineering. Mater. Sci. Eng. C.

[52] Li L., Lu C., Wang L., Chen M., White J.F., Hao X., McLean K.M., Chen H., Hughes T.C. (2018). Gelatin-Based Photocurable Hydrogels for Corneal Wound Repair. ACS Appl. Mater. Interfaces.

[53] Rizwan M., Peh G.S., Ang H.-P., Lwin N.C., Adnan K., Mehta J.S., Tan W.S., Yim E.K. (2017). Sequentially-crosslinked bioactive hydrogels as nano-patterned substrates with customizable stiffness and degradation for corneal tissue engineering applications. Biomaterials.

[54] Hartmann L., Watanabe K., Zheng L.L., Kim C.-Y., Beck S.E., Huie P., Noolandi J., Cochran J.R., Ta C.N., Frank C.W. (2011). Toward the development of an artificial cornea: Improved stability of interpenetrating polymer networks. J. Biomed. Mater. Res. Part B Appl. Biomater..

[55] Bigi A., Cojazzi G., Panzavolta S., Rubini K., Roveri N. (2001). Mechanical and thermal properties of gelatin films at different degrees of glutaraldehyde crosslinking. Biomaterials.

[56] Bigi A., Cojazzi G., Panzavolta S., Roveri N., Rubini K. (2002). Stabilization of gelatin films by crosslinking with genipin. Biomaterials.

[57] Yang C.-F., Yasukawa T., Kimura H., Miyamoto H., Honda Y., Tabata Y., Ikada Y., Ogura Y. (2000). Experimental Corneal Neovascularization by Basic Fibroblast Growth Factor Incorporated into Gelatin Hydrogel. Ophthalmic Res..

[58] Doillon C.J., Watsky M.A., Hakim M., Wang J., Munger R., Laycock N., Osborne R., Griffith M. (2003). A collagen-based scaf-fold for a tissue engineered human cornea: Physical and physiological properties. Int. J. Artif. Organs.

[59] Lu P.-L., Lai J.-Y., Ma D.H.-K., Hsiue G.-H. (2008). Carbodiimide cross-linked hyaluronic acid hydrogels as cell sheet delivery vehicles: Characterization and interaction with corneal endothelial cells. J. Biomater. Sci. Polym. Ed..

[60] Lai J.-Y., Ma D.H.-K., Cheng H.-Y., Sun C.-C., Huang S.-J., Li Y.-T., Hsiue G.-H. (2010). Ocular Biocompatibility of Carbodiimide Cross-Linked Hyaluronic Acid Hydrogels for Cell Sheet Delivery Carriers. J. Biomater. Sci. Polym. Ed..

[61] Bentley E., Murphy C.J., Li F., Carlsson D.J., Griffith M. (2010). Biosynthetic Corneal Substitute Implantation in Dogs. Cornea.

[62] Kitagawa K., Okabe M., Yanagisawa S., Zhang X.-Y., Nikaido T., Hayashi A. (2011). Use of a hyperdried cross-linked amniotic membrane as initial therapy for corneal perforations. Jpn. J. Ophthalmol..

[63] Calles J., Tártara L., López-García A., Diebold Y., Palma S., Vallés E. (2013). Novel bioadhesive hyaluronan–itaconic acid crosslinked films for ocular therapy. Int. J. Pharm..

[64] Lai J.-Y., Ma D.H.-K. (2013). Glutaraldehyde cross-linking of amniotic membranes affects their nanofibrous structures and limbal epithelial cell culture characteristics. Int. J. Nanomed..

[65] Nam E., Fujita N., Morita M., Tsuzuki K., Lin H.Y., Chung C.S., Nakagawa T., Nishimura R. (2014). Comparison of the canine corneal epithelial cell sheets cultivated from limbal stem cells on canine amniotic membrane, atelocollagen gel, and temperature-responsive culture dish. Veter Ophthalmol..

[66] Yu S., Zhang X., Tan G., Tian L., Liu D., Liu Y., Yang X., Pan W. (2017). A novel pH-induced thermosensitive hydrogel composed of carboxymethyl chitosan and poloxamer cross-linked by glutaraldehyde for ophthalmic drug delivery. Carbohydr. Polym..

[67] Rafat M., Griffith M., Hakim M., Muzakare L., Li F., Khulbe K., Matsuura T. (2007). Plasma surface modification and characterization of collagen-based artificial cornea for enhanced epithelialization. J. Appl. Polym. Sci..

[68] Nicoletti A., Fiorini M., Paolillo J., Dolcini L., Sandri M., Pressato D. (2012). Effects of different crosslinking conditions on the chemical–physical properties of a novel bio-inspired composite scaffold stabilised with 1,4-butanediol diglycidyl ether (BDDGE). J. Mater. Sci. Mater. Med..

[69] Wang S., Liu W., Han B., Yang L. (2009). Study on a hydroxypropyl chitosan–gelatin based scaffold for corneal stroma tissue engineering. Appl. Surf. Sci..

[70] Koh L.B., Islam M.M., Mitra D., Noel C.W., Merrett K., Odorcic S., Fagerholm P., Jackson W.B., Liedberg B., Phopase J. (2013). Epoxy Cross-Linked Collagen and Collagen-Laminin Peptide Hydrogels as Corneal Substitutes. J. Funct. Biomater..

[71] Butler M.F., Ng Y.-F., Pudney P.D.A. (2003). Mechanism and kinetics of the crosslinking reaction between biopolymers containing primary amine groups and genipin. J. Polym. Sci. Part A Polym. Chem..

[72] Tonda-Turo C., Gentile P., Saracino S., Chiono V., Nandagiri V., Muzio G., Canuto R., Ciardelli G. (2011). Comparative analysis of gelatin scaffolds crosslinked by genipin and silane coupling agent. Int. J. Biol. Macromol..

[73] Madhavan K., Belchenko D., Motta A., Tan W. (2010). Evaluation of composition and crosslinking effects on collagen-based composite constructs. Acta Biomater..

[74] Fessel G., Cadby J., Wunderli S., van Weeren R., Snedeker J.G. (2014). Dose- and time-dependent effects of genipin crosslinking on cell viability and tissue mechanics—Toward clinical application for tendon repair. Acta Biomater..

[75] Grolik M., Szczubiałka K., Wowra B., Dobrowolski D., Orzechowska-Wylęgała B., Wylęgała E., Nowakowska M. (2012). Hydrogel membranes based on genipin-cross-linked chitosan blends for corneal epithelium tissue engineering. J. Mater. Sci. Mater. Med..

[76] Li Y.-H., Cheng C.-Y., Wang N.-K., Tan H.-Y., Tsai Y.-J., Hsiao C.-H., Ma D.H.-K., Yeh L.-K. (2015). Characterization of the modified chitosan membrane cross-linked with genipin for the cultured corneal epithelial cells. Colloids Surf. B Biointerfaces.

[77] Yu Y., Feng R., Li J., Wang Y., Song Y., Tan G., Liu D., Liu W., Yang X., Pan H. (2019). A hybrid genipin-crosslinked dual-sensitive hydrogel/nanostructured lipid carrier ocular drug delivery platform. Asian J. Pharm. Sci..

[78] Nam K., Kimura T., Kishida A. (2008). Controlling Coupling Reaction of EDC and NHS for Preparation of Collagen Gels Using Ethanol/Water Co-Solvents. Macromol. Biosci..

[79] Cammarata C.R., Hughes M.E., Ofner C.M. (2015). Carbodiimide Induced Cross-Linking, Ligand Addition, and Degradation in Gelatin. Mol. Pharm..

[80] Bax D.V., Davidenko N., Gullberg D., Hamaia S.W., Farndale R.W., Best S.M., Cameron R.E. (2017). Fundamental insight into the effect of carbodiimide crosslinking on cellular recognition of collagen-based scaffolds. Acta Biomater..

[81] Damink L.O., Dijkstra P.J., Van Luyn M., Van Wachem P., Nieuwenhuis P., Feijen J. (1996). Cross-linking of dermal sheep collagen using a water-soluble carbodiimide. Biomaterials.

[82] Pieper J., Hafmans T., Veerkamp J., Van Kuppevelt T. (2000). Development of tailor-made collagen–glycosaminoglycan matrices: EDC/NHS crosslinking, and ultrastructural aspects. Biomaterials.

[83] Davidenko N., Schuster C., Bax D., Raynal N., Farndale R., Best S., Cameron R. (2015). Control of crosslinking for tailoring collagen-based scaffolds stability and mechanics. Acta Biomater..

[84] Liu W., Merrett K., Griffith M., Fagerholm P., Dravida S., Heyne B., Scaiano J.C., Watsky M.A., Shinozaki N., Lagali N. (2008). Recombinant human collagen for tissue engineered corneal substitutes. Biomaterials.

[85] Rafat M., Li F., Fagerholm P., Lagali N.S., Watsky M.A., Munger R., Matsuura T., Griffith M. (2008). PEG-stabilized carbodiimide crosslinked collagen–chitosan hydrogels for corneal tissue engineering. Biomaterials.

[86] Liu W., Deng C., McLaughlin C.R., Fagerholm P., Lagali N.S., Heyne B., Scaiano J.C., Watsky M.A., Kato Y., Munger R. (2009). Collagen–phosphorylcholine interpenetrating network hydrogels as corneal substitutes. Biomaterials.

[87] Ma D.H.-K., Lai J.-Y., Cheng H.-Y., Tsai C.-C., Yeh L.-K. (2010). Carbodiimide cross-linked amniotic membranes for cultivation of limbal epithelial cells. Biomaterials.

[88] Lai J.-Y. (2016). Hyaluronic acid concentration-mediated changes in structure and function of porous carriers for corneal endothelial cell sheet delivery. Mater. Sci. Eng. C.

[89] Lai J.Y., Cheng H.Y., Ma D.H.K. (2015). Investigation of overrun-processed porous hyaluronic acid carriers in corneal endo-thelial tissue engineering. PLoS ONE.

[90] Goodarzi H., Jadidi K., Pourmotabed S., Sharifi E., Aghamollaei H. (2019). Preparation and in vitro characterization of cross-linked collagen–gelatin hydrogel using EDC/NHS for corneal tissue engineering applications. Int. J. Biol. Macromol..

[91] Duan X., Sheardown H. (2006). Dendrimer crosslinked collagen as a corneal tissue engineering scaffold: Mechanical properties and corneal epithelial cell interactions. Biomaterials.

[92] Myung D., Koh W., Bakri A., Zhang F., Marshall A., Ko J., Noolandi J., Carrasco M., Cochran J.R., Frank C.W. (2007). Design and fabrication of an artificial cornea based on a photolithographically patterned hydrogel construct. Biomed. Microdevices.

[93] Farooqui N., Myung D., Koh W., Masek R., Dalal M., Carrasco M.R., Noolandi J., Frank C.W., Ta C.N. (2007). Histological processing of ph-sensitive hydrogels used in corneal implant applications. J. Histotechnol..

[94] Myung D., Farooqui N., Zheng L.L., Koh W., Gupta S., Bakri A., Noolandi J., Cochran J.R., Frank C.W., Ta C.N. (2009). Bioactive interpenetrating polymer network hydrogels that support corneal epithelial wound healing. J. Biomed. Mater. Res. Part A.

[95] Merrett K., Liu W., Mitra D., Camm K.D., McLaughlin C.R., Liu Y., Watsky M.A., Li F., Griffith M., Fogg D.E. (2009). Syn-thetic neoglycopolymer-recombinant human collagen hybrids as biomimetic crosslinking agents in corneal tissue engineering. Biomaterials.

[96] Liang Y., Liu W., Han B., Yang C., Ma Q., Song F., Bi Q. (2011). An in situ formed biodegradable hydrogel for reconstruction of the corneal endothelium. Colloids Surf. B Biointerfaces.

[97] Park S., Nam S.H., Koh W.-G. (2011). Preparation of collagen-immobilized poly(ethylene glycol)/poly(2-hydroxyethyl methacrylate) interpenetrating network hydrogels for potential application of artificial cornea. J. Appl. Polym. Sci..

[98] Ozcelik B., Brown K.D., Blencowe A., Daniell M., Stevens G.W., Qiao G.G. (2013). Ultrathin chitosan–poly(ethylene glycol) hydrogel films for corneal tissue engineering. Acta Biomater..

[99] Xiang J., Sun J., Hong J., Wang W., Wei A., Le Q., Xu J. (2015). T-style keratoprosthesis based on surface-modified poly (2-hydroxyethyl methacrylate) hydrogel for cornea repairs. Mater. Sci. Eng. C.

[100] Lei L., Li X., Xiong T., Yu J., Yu X., Song Z., Li X. (2018). Covalently Cross-Linked Chitosan Hydrogel Sheet for Topical Ophthalmic Delivery of Levofloxacin. J. Biomed. Nanotechnol..

[101] Sani E.S., Kheirkhah A., Rana D., Sun Z.M., Foulsham W., Sheikhi A., Khademhosseini A., Dana R., Annabi N. (2019). Su-tureless repair of corneal injuries using naturally derived bioadhesive hydrogels. Sci. Adv..

[102] Lai J.-Y. (2012). Biocompatibility of Genipin and Glutaraldehyde Cross-Linked Chitosan Materials in the Anterior Chamber of the Eye. Int. J. Mol. Sci..

[103] Ahn J.-I., Kuffova L., Merrett K., Mitra D., Forrester J.V., Li F., Griffith M. (2013). Crosslinked collagen hydrogels as corneal implants: Effects of sterically bulky vs. non-bulky carbodiimides as crosslinkers. Acta Biomater..

[104] Wang K., Han Z. (2017). Injectable hydrogels for ophthalmic applications. J. Control. Release.

[105] Overstreet D.J., Dutta D., Stabenfeldt S.E., Vernon B.L. (2012). Injectable hydrogels. J. Polym. Sci. Part B Polym. Phys..

[106] Li Y.L., Rodrigues J., Tomas H. (2012). Injectable and biodegradable hydrogels: Gelation, biodegradation and biomedical ap-plications. Chem. Soc. Rev..

[107] Yang J.-A., Yeom J., Hwang B.W., Hoffman A.S., Hahn S.K. (2014). In situ-forming injectable hydrogels for regenerative medicine. Prog. Polym. Sci..

[108] Hoffman A.S. (2002). Hydrogels for biomedical applications. Adv. Drug Deliv. Rev..

[109] Ulijn R.V., Bibi N., Jayawarna V., Thornton P.D., Todd S.J., Mart R.J., Smith A.M., Gough J.E. (2007). Bioresponsive hydro-gels. Mater. Today.

[110] Xinming L., Yingde C., Lloyd A.W., Mikhalovsky S.V., Sandeman S.R., Howel C.A., Liewen L. (2008). Polymeric hydrogels for novel contact lens-based ophthalmic drug delivery systems: A review. Contact Lens Anterior Eye.

[111] Chien Y., Liao Y.W., Liu D.M., Lin H.L., Chen S.J., Chen H.L., Peng C.H., Liang C.M., Mou C.Y., Chiou H. (2012). Corneal repair by human corneal keratocyte-reprogrammed ipscs and amphiphatic carboxymethyl-hexanoyl chitosan hy-drogel. Biomaterials.

[112] Garagorri N., Fermanian S., Thibault R., Ambrose W.M., Schein O.D., Chakravarti S., Elisseeff J. (2008). Keratocyte behavior in three-dimensional photopolymerizable poly(ethylene glycol) hydrogels. Acta Biomater..

[113] McTiernan C.D., Simpson F.C., Haagdorens M., Samarawickrama C., Hunter D., Buznyk O., Fagerholm P., Ljunggren M.K., Lewis P., Pintelon I. (2020). LiQD Cornea: Pro-regeneration collagen mimetics as patches and alternatives to corneal transplantation. Sci. Adv..

[114] Anumolu S.S., DeSantis A.S., Menjoge A.R., Hahn R.A., Beloni J.A., Gordon M.K., Sinko P.J. (2010). Doxycycline loaded poly(ethylene glycol) hydrogels for healing vesicant-induced ocular wounds. Biomaterials.

[115] Pratoomsoot C., Tanioka H., Hori K., Kawasaki S., Kinoshita S., Tighe P.J., Dua H., Shakesheff K.M., Rose F.R.A. (2008). A thermoreversible hydrogel as a biosynthetic bandage for corneal wound repair. Biomaterials.

[116] Fanesi G., Abrami M., Zecchin F., Giassi I., Dal Ferro E., Boisen A., Grassi G., Bertoncin P., Grassi M., Marizza P. (2018). Com-bined used of rheology and lf-nmr for the characterization of pvp-alginates gels containing liposomes. Pharm. Res..

[117] Ahearne M., Yang Y., El Haj A.J., Then K.Y., Liu K.-K. (2005). Characterizing the viscoelastic properties of thin hydrogel-based constructs for tissue engineering applications. J. R. Soc. Interface.

[118] Wen J.H., Vincent L.G., Fuhrmann A., Choi Y.S., Hribar K.C., Taylor-Weiner H., Chen S., Engler A.J. (2014). Interplay of matrix stiffness and protein tethering in stem cell differentiation. Nat. Mater..

[119] Ahearne M., Wilson S.L., Liu K.-K., Rauz S., El Haj A.J., Yang Y. (2010). Influence of cell and collagen concentration on the cell–matrix mechanical relationship in a corneal stroma wound healing model. Exp. Eye Res..

[120] Li X., Zhang J., Kawazoe N., Chen G. (2017). Fabrication of highly crosslinked gelatin hydrogel and its influence on chondro-cyte proliferation and phenotype. Polymers.

[121] Kharkar P.M., Kiick K.L., Kloxin A.M. (2013). Designing degradable hydrogels for orthogonal control of cell microenviron-ments. Chem. Soc. Rev..

[122] Bryant S.J., Bender R.J., Durand K.L., Anseth K.S. (2004). Encapsulating chondrocytes in degrading PEG hydrogels with high modulus: Engineering gel structural changes to facilitate cartilaginous tissue production. Biotechnol. Bioeng..

[123] Li X., Sun Q., Li Q., Kawazoe N., Chen G. (2018). Functional hydrogels with tunable structures and properties for tissue engi-neering applications. Front. Chem..

[124] Richbourg N.R., Peppas N.A. (2020). The swollen polymer network hypothesis: Quantitative models of hydrogel swelling, stiffness, and solute transport. Prog. Polym. Sci..

[125] Dai W., Barbari T. (1999). Hydrogel membranes with mesh size asymmetry based on the gradient crosslinking of poly(vinyl alcohol). J. Membr. Sci..

[126] Peppas N.A., Hilt J.Z., Khademhosseini A., Langer R. (2006). Hydrogels in Biology and Medicine: From Molecular Principles to Bionanotechnology. Adv. Mater..

[127] Aimetti A.A., Machen A.J., Anseth K.S. (2009). Poly(ethylene glycol) hydrogels formed by thiol-ene photopolymerization for enzyme-responsive protein delivery. Biomaterials.

[128] Hu W., Wang Z., Xiao Y., Zhang S., Wang J. (2019). Advances in crosslinking strategies of biomedical hydrogels. Biomater. Sci..

[129] Lu S., Lam J., Trachtenberg J.E., Lee E.J., Seyednejad H., van den Beucken J.J.J.P., Tabata Y., Wong M.E., Jansen J.A., Mikos A.G. (2014). Dual growth factor delivery from bilayered, biodegradable hydrogel composites for spatially-guided osteochondral tissue repair. Biomaterials.

[130] Hadjipanayi E., Ananta M., Binkowski M., Streeter I., Lu Z., Cui Z.F., Brown R.A., Mudera V. (2010). Mechanisms of structure generation during plastic compression of nanofibrillar collagen hydrogel scaffolds: Towards engineering of collagen. J. Tissue Eng. Regen. Med..

[131] Cheema U., Brown R.A. (2013). Rapid Fabrication of Living Tissue Models by Collagen Plastic Compression: Understanding Three-Dimensional Cell Matrix Repair In Vitro. Adv. Wound Care.

[132] Torbet J., Ronzière M.C. (1984). Magnetic alignment of collagen during self-assembly. Biochem. J..

[133] Torbet J., Malbouyres M., Builles N., Justin V., Roulet M., Damour O., Oldberg A., Ruggiero F., Hulmes D.J. (2007). Orthogo-nal scaffold of magnetically aligned collagen lamellae for corneal stroma reconstruction. Biomaterials.

[134] Panteli P.A., Patrickios C.S. (2019). Multiply Interpenetrating Polymer Networks: Preparation, Mechanical Properties, and Applications. Gels.

[135] Krishnakumar G.S., Sampath S., Muthusamy S., John M.A. (2019). Importance of crosslinking strategies in designing smart biomaterials for bone tissue engineering: A systematic review. Mater. Sci. Eng. C.

[136] Martínez A., Blanco M., Davidenko N., Cameron R. (2015). Tailoring chitosan/collagen scaffolds for tissue engineering: Effect of composition and different crosslinking agents on scaffold properties. Carbohydr. Polym..

[137] Khor E. (1997). Methods for the treatment of collagenous tissues for bioprostheses. Biomaterials.

[138] Casali D.M., Yost M.J., Matthews M.A. (2018). Eliminating glutaraldehyde from crosslinked collagen films using supercritical CO2. J. Biomed. Mater. Res. Part A.

[139] Yamazaki M., Chiba K., Mohri T., Hatanaka H. (2008). Activation of the mitogen-activated protein kinase cascade through nitric oxide synthesis as a mechanism of neuritogenic effect of genipin in PC12h cells. J. Neurochem..

[140] Kim B.-C., Kim H.-G., Lee S.-A., Lim S., Park E.-H., Kim S.-J., Lim C.-J. (2005). Genipin-induced apoptosis in hepatoma cells is mediated by reactive oxygen species/c-Jun NH2-terminal kinase-dependent activation of mitochondrial pathway. Biochem. Pharmacol..

[141] Kirchhof S., Goepferich A.M., Brandl F.P. (2015). Hydrogels in ophthalmic applications. Eur. J. Pharm. Biopharm..

[142] Hammer N., Brandl F.P., Kirchhof S., Messmann V., Goepferich A.M. (2015). Protein Compatibility of Selected Cross-linking Reactions for Hydrogels. Macromol. Biosci..

[143] Zhang B., Xue Q., Li J., Ma L., Yao Y., Ye H., Cui Z., Yang H. (2019). 3d bioprinting for artificial cornea: Challenges and perspectives. Med. Eng. Phys..

[144] Sorkio A., Koch L., Koivusalo L., Deiwick A., Miettinen S., Chichkov B., Skottman H. (2018). Human stem cell based corneal tissue mimicking structures using laser-assisted 3D bioprinting and functional bioinks. Biomaterials.

[145] Isaacson A., Swioklo S., Connon C.J. (2018). 3D bioprinting of a corneal stroma equivalent. Exp. Eye Res..

[146] Campos D.F.D., Rohde M., Ross M., Anvari P., Blaeser A., Vogt M., Panfil C., Yam G.H., Mehta J.S., Fischer H. (2019). Corneal bioprinting utilizing collagen-based bioinks and primary human keratocytes. J. Biomed. Mater. Res. Part A.

